# Characterization of Antibiotic-Tolerant Persister Cells in *Mannheimia haemolytica*

**DOI:** 10.3390/ani16162610

**Published:** 2026-08-20

**Authors:** Qibing Gu, Zhijun Chen, Taichun Gao, Linbo Zhao, Guangfu Zhao, Falong Yang

**Affiliations:** College of Animal & Veterinary Sciences, Southwest Minzu University, Chengdu 610041, China; guqbing@swun.edu.cn (Q.G.); czj0312@outlook.com (Z.C.); 17808341353@163.com (T.G.); zhaolb15233930215@163.com (L.Z.); zgf515005117@outlook.com (G.Z.)

**Keywords:** *Mannheimia haemolytica*, persister cells, antibiotic tolerance, stress, eradication

## Abstract

Antibiotics are the primary treatment for bacterial respiratory diseases in ruminants. *Mannheimia haemolytica* is the major etiological agent causing respiratory diseases in ruminants, but its tolerance to antibiotics remains poorly understood. In this study, strains of *M. haemolytica* belonging to the main serotypes A1, A2, and A6 were selected for analysis of their ability to form multidrug-tolerant persister cells. The results showed that *M. haemolytica* was able to form approximately 10^4^ CFU/mL of antibiotic-tolerant persisters during both the logarithmic phase and the stationary phase. Compared to the logarithmic phase, the ability of stationary phase cells to form persisters was significantly enhanced. Further analysis revealed that different stress conditions had varying effects on the ability of the three serotype strains to form persister cells. Under low dissolved oxygen conditions, the ability of the three serotype strains to form multidrug-tolerant persisters was significantly weakened. Oxidative stress conditions exerted the greatest effect on persister formation in the serotype A2 strain QS2-1, whereas acid stress had the greatest impact on persister formation in the serotype A6 strain Mh1128. Finally, strategies for the eradication of *M. haemolytica* persister cells were examined. The use of antibiotic combinations with distinct mechanisms of action can effectively and rapidly reduce the formation of persister populations in certain strains under our experimental conditions. Furthermore, L-Serine and mannitol were shown to enhance the susceptibility of most strains to florfenicol. Overall, this study examined the capacity of *M. haemolytica* to form persisters and explored the factors influencing the formation of these persisters, as well as potential strategies for their eradication. The findings offer a theoretical foundation for the treatment of *M. haemolytica* respiratory infections in ruminant species.

## Simple Summary

The use of antibiotics is the primary treatment for respiratory diseases in cattle and sheep. However, bacteria can develop tolerance to antibiotics by forming persister cells, leading to treatment failure. As a major pathogen causing respiratory diseases in ruminants, the tolerance of *Mannheimia haemolytica* to commonly used clinical antibiotics remains unclear. To investigate the ability of *M. haemolytica* to form persisters, this study analyzed persister formation in three serotypes of the bacterium. The findings indicated that following a 12 h exposure to high concentrations of antibiotics, *M. haemolytica* was capable of generating a persister population estimated at approximately 10^4^ CFU/mL. Different stress conditions have varying effects on the formation of persisters. The combined use of different antibiotics can effectively reduce the formation of some *M. haemolytica* persisters. This study provides a theoretical basis for the treatment of *M. haemolytica* infections in ruminants.

## 1. Introduction

Respiratory diseases are the main cause of morbidity, mortality, and reduced production performance in ruminants, resulting in significant economic losses to the livestock industry each year [1,2,3]. The main bacterial pathogen causing respiratory diseases in ruminants is *Mannheimia haemolytica* [4]. *M. haemolytica* is a weakly hemolytic, Gram-negative opportunistic pathogen that commonly colonizes the upper respiratory tract of ruminants and invades the lower respiratory tract when animals are infected with viruses, physiological stress, or have impaired immune function [5,6]. *M. haemolytica*, as a ruminant pathogen, is associated not only with severe respiratory diseases in domestic sheep, bighorn sheep, and cattle, but also with lamb septicemia and ewe mastitis [7,8,9]. Based on differences in capsular antigens, *M. haemolytica* bacteria are currently classified into 12 serotypes (A1, A2, A5–A9, A12–A14, A16, and A17) [10]. Among them, serotypes A1, A2, and A6 are the most common isolates from the respiratory tract of ruminants [10,11]. Facilitates the adhesion and colonization of *M. haemolytica* on the mucosal surfaces of the upper respiratory tract via various surface adhesins [12,13]. In addition, *M. haemolytica* also produces various virulence factors that evade host defenses and promote the development of pneumonia [14].

Previous studies have reported multidrug-resistant and even extensively drug-resistant phenotypes of *M. haemolytica* in ruminants [15,16,17]. Nevertheless, the antimicrobial susceptibility patterns of *M. hemolytica* vary significantly depending on geographic region, time of isolation, and the host species involved [18,19]. The main treatment for bacterial respiratory diseases in ruminants is antibiotics [20]. Although there has been much research on *M. haemolytica* vaccines, these vaccines have poor protective effects or cause more severe symptoms [14]. Therefore, antibiotics are particularly important in the treatment of *M. haemolytica* infections. Due to the extensive use and abuse of antibiotics in clinical practice, resistance to *M. haemolytica* has become increasingly severe [20]. Of particular significance, bacteria such as *M. haemolytica*, members of the *Pasteurellaceae* family, harbor mobile genetic elements that encode multiple antibiotic resistance genes [21,22,23,24]. These genetic elements are capable of horizontal transfer between *M. haemolytica* and *Pasteurella multocida*, thereby facilitating the dissemination of antimicrobial resistance [25]. Therefore, the decline in antibiotic sensitivity of *M. haemolytica* warrants further attention.

Persister cells, which represent antibiotic-tolerant phenotypic variants, play a critical role in the emergence and progression of bacterial resistance [26]. The existence of persisters not only extends the duration of clinical antibiotic administration and elevates treatment expenses but also heightens the risk of bacterial resistance development. Therefore, the development of novel antimicrobial therapies that reduce the formation of persister cells has gradually become a new strategy for treating bacterial infections. At present, multiple bacterial species have been identified as capable of forming persister cells. These include Gram-negative bacteria such as *Klebsiella pneumoniae*, *Salmonella* typhimurium, and *Pseudomonas aeruginosa*, in addition to Gram-positive bacteria including *Staphylococcus aureus* and *Streptococcus suis* [27,28,29,30,31]. As an important pathogen causing respiratory diseases in ruminants, the ability of *M. haemolytica* to form persisters has not yet been investigated. Investigations into *M. haemolytica* persisters are expected to advance the development of therapeutic strategies for respiratory diseases in ruminant species.

In this study, we investigated the ability of *M. haemolytica* strains of the predominant serotypes A1, A2, and A6 to form multidrug-tolerant persisters. Concurrently, we examined the capacity of *M. haemolytica* to generate persister cells when subjected to various pressure-induced stress conditions. Finally, this study further explored strategies for eliminating *M. haemolytica* persisters, providing a theoretical basis for the treatment of bacterial respiratory diseases in ruminants.

## 2. Materials and Methods

### 2.1. Bacterial Strains and Growth Conditions

All *M. haemolytica* strains were stored at −80 °C in our laboratory. Detailed information on the strains used in this study is provided in Table S1. All strains were isolated from animals exhibiting respiratory symptoms, including cough, dyspnea, nasal discharge, and fever. The A1 serotype strains S19, S10-1, Mh1202, and S30 were isolated from nasal swab samples collected from various sheep. Serotype A2 strains QS2-1, D16-1, and S235 were isolated from nasal swabs taken from different individual sheep, while serotype A6 strain Mh1128 was isolated from a nasal swab sample taken from a goat. The identification of serotypes of *M. haemolytica* was based on methods reported in the literature [32]. The primers utilized for the identification of *M. haemolytica* and its respective serotypes are detailed in Table S2. Specifically, the MropB-F and MropB-R primers are employed for the detection of *M. haemolytica*, whereas the MhA1-F/R, MhA2-F/R, and MhA6-F/R primers are designated for the identification of serotypes A1, A2, and A6, respectively. *M. haemolytica* was cultured on Trypticase Soy Agar (TSA, HaiBo Biotechnology Co., Ltd, Qingdao, China) or Trypticase Soy Broth (TSB, HaiBo Biotechnology Co., Ltd, Qingdao, China) medium at 37 °C. The strains were resuscitated on TSA plates containing 5% defibrinated sheep blood. The strains were grown in TSB liquid culture (180 rpm, 37 °C).

### 2.2. Antimicrobial Susceptibility Tests

The minimum inhibitory concentrations (MICs) of antibiotics against *M. haemolytica* were determined according to guidelines established by the Clinical and Laboratory Standards Institute [33]. In ruminant livestock production, antibiotics including β-lactams, tetracyclines, fluoroquinolones, and amphenicols are frequently employed to manage bacterial respiratory infections [34]. Consequently, the antibiotics ampicillin and ceftiofur (β-lactams), tetracycline and terramycin (tetracyclines), enrofloxacin (fluoroquinolones), and florfenicol (amphenicols) were chosen for detailed investigation. *M. haemolytica* was cultured to the logarithmic phase then diluted 1000-fold with fresh culture medium. Add 20 μL of antibiotics to the first well of the 96-well plate and mix thoroughly, then transfer 100 μL of the mixture to the subsequent wells until the 10th well. The 11th and 12th wells were used as positive and negative controls, respectively. Observe and record the results after incubating at 37 °C for 20 h.

### 2.3. Persister Cell Formation Assay

The determination of persisters of *M. haemolytica* was performed according to the method described in the literature [31]. Drawing upon existing literature and considering the solubility properties of the antibiotics employed, this study established a treatment concentration of 50 × MIC and an exposure period of 12 h. Inoculate *M. haemolytica* on TSA plates containing 5% defibrinated sheep blood, and pick up single colonies on the second day for expansion culture. Overnight cultures of *M. haemolytica* were transferred to fresh culture medium and diluted to 1 × 10^8^ CFU/mL after the bacteria reached the logarithmic or stationary phase. Then add 50 times MIC of the antibiotic and incubate for 12 h. Take samples at 0, 4, 8, and 12 h, and determine CFU by continuous dilution plate count. At each designated time point, the collected samples were washed with sterile PBS to eliminate any remaining antibiotics from the culture medium. After incubating the plates at 37 °C for 24 h, count the CFUs. All antibiotic killing tests should be performed with at least three biological replicates, with each test conducted independently three times.

### 2.4. Persistence Phenotype Analysis

After *M. haemolytica* has grown to a stationary phase (Generation 1), dilute it to a concentration of 1 × 10^8^ CFU/mL. Then add 50 times MIC antibiotics, incubate for 8 h, and determine the formation of persisters. Collect the bacteria that survive after 8 h, wash them with sterile PBS, and reculture them until they reach a stationary phase (Generation 2). Subsequently, high-concentration antibiotic treatment was administered again to observe the formation of persisters. Re-culture the bacteria (Generation 3) that survived for 8 h in the generation 2 and perform the third round of antibiotic treatment and persister analysis. Analyze the heritability of persistence phenotypes based on the formation of persisters in three generations of *M. haemolytica*.

### 2.5. Pressure Stress Analysis of Persister Formation

In the ruminant respiratory tract, *M. haemolytica* encounters microenvironments with reduced oxygen tension. During pneumonia, alveolar consolidation, accumulation of inflammatory exudate, and mucus plugging create localized hypoxic zones within the infected lung tissue [35]. Studies on other Gram-negative pathogens have demonstrated that low oxygen can significantly influence persister cell formation [36]. Consequently, examining the impact of low dissolved oxygen on persister cell formation in *M. haemolytica* is highly pertinent to the physiological conditions this pathogen experiences within the host during clinical respiratory infections. Conduct low dissolved oxygen tests in accordance with the literature and any necessary modifications [37]. In the low dissolved-oxygen test, *M. haemolytica* in the growth-stationary phase was diluted to 1 × 10^8^ CFU/mL. Five milliliters of the bacterial solution were transferred to a 10 mL round-bottom centrifuge tube as the control group, while five milliliters of the bacterial solution were transferred to a 5 mL round-bottom centrifuge tube as the experimental group. Both the control group and the experimental group were treated with high concentrations (50 × MIC) of tetracycline, florfenicol, ampicillin, and ceftiofur. Samples were collected at 0 h and 8 h for dilution and plate counting.

In the ruminant respiratory tract, multiple acidic microenvironments exist that are associated with *M. haemolytica* infection. Inflammatory lesions in pneumonic lung tissue frequently demonstrate a reduced pH, attributable to the accumulation of lactic acid generated through anaerobic glycolysis by infiltrating host cells and the fermentative metabolism of bacteria [38]. Furthermore, following internalization by alveolar macrophages, *M. haemolytica* may encounter an acidic environment within the phagolysosomes [39]. Therefore, we further analyzed the effect of acid stress on persister cell formation in *M. haemolytica*. According to the reference and its modifications [40], during the acid stress test, the pH of the culture medium in the experimental group was adjusted to 6.5, while the control group was maintained at normal pH levels. Both the control group and the experimental group were filled with 5 mL of bacterial solution in 10 mL EP tubes. Subsequently, the four high-concentration antibiotics were used for treatment, and samples were taken at 0, 4, 8, and 12 h to measure the persister cells.

In ruminants, alveolar macrophages and recruited neutrophils generate substantial quantities of reactive oxygen species (ROS), such as superoxide anion and hydrogen peroxide, which play a critical role in the innate immune defense [41]. Previous studies have demonstrated that *M. haemolytica* is capable of surviving the oxidative burst during phagocytosis and persisting within inflamed lung tissue where ROS concentrations are elevated [42]. To examine the impact of oxidative stress on the formation of persistent cells in *M. haemolytica*, we employed hydrogen peroxide treatment. Oxidative stress experiments were conducted according to the reference [43], with a final concentration of hydrogen peroxide of 0.1 mM in the culture medium of the experimental group. Both the control group and the experimental group were also filled with 5 mL of bacterial solution in 10 mL EP tubes. Similarly, four types of high-concentration antibiotics were used to treat and measure the persister cells.

### 2.6. Persister Cell Elimination Test

First, a combination of two antibiotics with different mechanisms of action was used: ampicillin and enrofloxacin, ampicillin and kanamycin, or enrofloxacin and kanamycin. Secondly, L-serine was added to the experimental group at a final concentration of 15 mM [44], or Mannitol was added at a final concentration of 40 mM [45]. The method for determining persister cells is described above.

### 2.7. Statistical Analyses

Each experiment was conducted a minimum of three times to ensure reproducibility. Data analysis and visualization were conducted using GraphPad Prism version 8. Data on the proportion of persisters among single-time-point treatment groups and control groups under low dissolved oxygen, acid stress, and oxidative stress were analyzed using an unpaired two-tailed Student’s *t*-test. The analysis of complete time-kill curves for identifying persisters of *M. haemolytica* under different antibiotics was performed using the log-rank test (Mantel–Cox). A threshold of *p* < 0.05 was established to determine statistical significance.

## 3. Results

### 3.1. Identification of Multidrug-Tolerant Persisters in M. haemolytica

**Figure 1:**
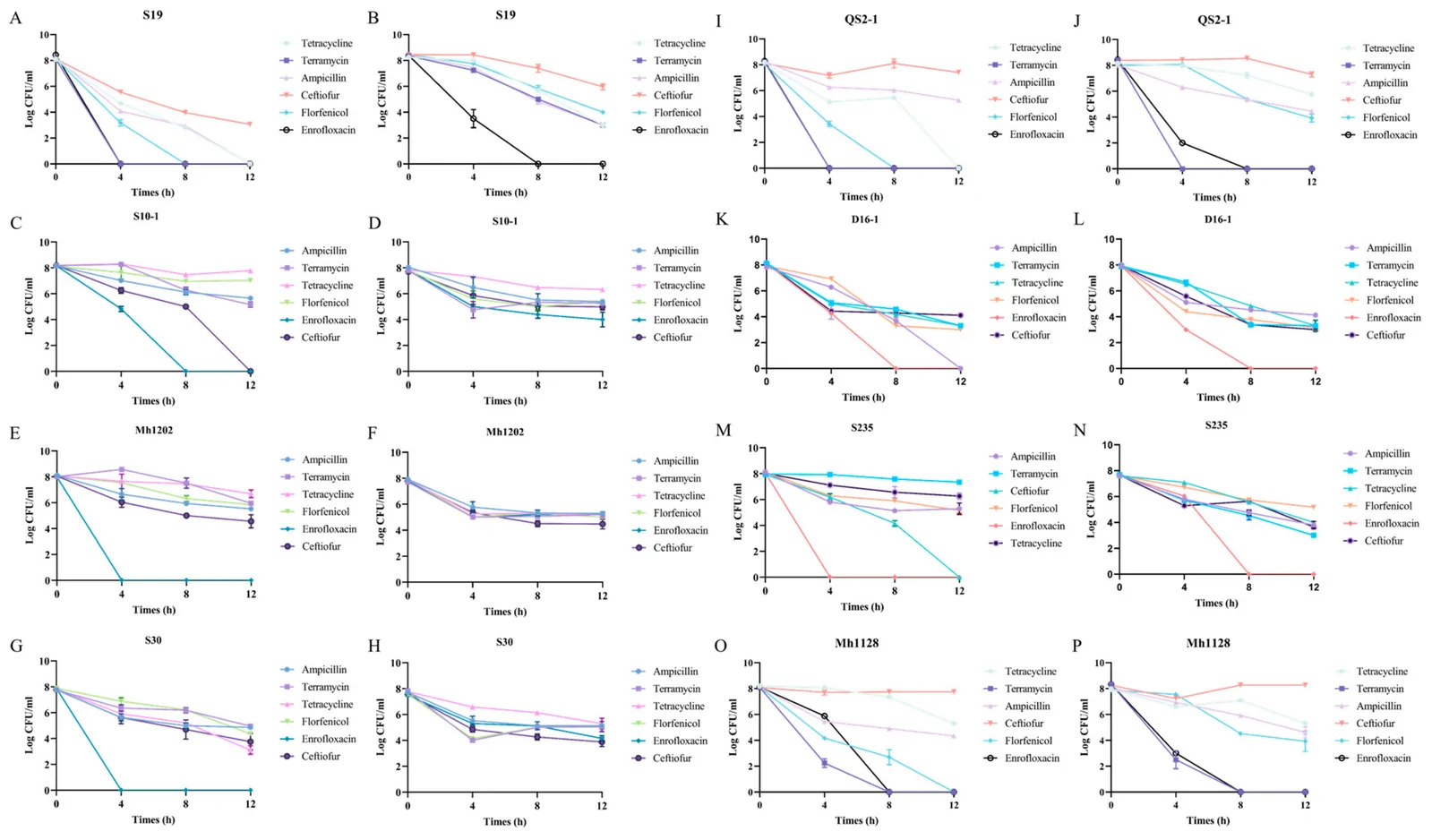
Analysis of persister formation in *M. haemolytica* under different antibiotics during the stationary and logarithmic phases. Logarithmic-grown *M. haemolytica* strains S19 (**A**), S10-1 (**C**), Mh1202 (**E**), S30 (**G**), QS2-1 (**I**), D16-1 (**K**), S235 (**M**), and Mh1128 (**O**) were treated with 50-fold MICs of tetracycline, terramycin, ampicillin, ceftiofur, florfenicol, and enrofloxacin antibiotics for 12 h. Stationary-grown strains S19 (**B**), S10-1 (**D**), Mh1202 (**F**), S30 (**H**), QS2-1 (**J**), D16-1 (**L**), S235 (**N**), and Mh1128 (**P**) were treated with 50-fold MICs of antibiotics for 12 h. At the indicated time points, CFUs were determined.

The formation of persisters not only promotes persistent and recurrent clinical infections but also plays a crucial role in the development of bacterial resistance. To investigate the ability of *M. haemolytica* to form persister cells, we further selected the clinically predominant serotypes A1 (S19, S10-1, Mh1202, S30), A2 (QS2-1, D16-1, S235), and A6 (Mh1128) strains (Figure S1). First, we determined the MICs of different antibiotics against *M. haemolytica* (Table 1). Subsequently, the ability of *M. haemolytica* in the logarithmic and stationary phases to form persisters under 50-fold MIC antibiotics was analyzed. The findings indicated that during the logarithmic growth phase, the S19 strain exhibited tolerance exclusively to ceftiofur, whereas all other tested antibiotics were effective in eradicating the bacterial population (Figure 1A). During the stationary phase, the formation levels of persisters tolerant to tetracycline, terramycin, ampicillin, ceftiofur, and florfenicol in the S19 strain increased significantly compared to the logarithmic phase (Figure 1B). In the other three serotype A1 strains, S10-1 (Figure 1C,D), Mh1202 (Figure 1E,F), and S30 (Figure 1G,H), tolerance to tetracycline, terramycin, ampicillin, ceftiofur, and florfenicol was observed in both the logarithmic and stationary phases. Furthermore, the stationary phase significantly increased the formation levels of enrofloxacin-tolerant persisters in these strains. The QS2-1 strain produced approximately 10^4^ to 10^7^ CFU/mL of ampicillin and ceftiofur-tolerant persisters during both the logarithmic and stationary phases, while during the stationary phase, there was a significant increase in persisters tolerant to tetracycline and florfenicol (Figure 1I,J). For strains D16-1 (Figure 1K,L) and S235 (Figure 1M,N), the formation of ampicillin- and ceftiofur-tolerant persisters increased in the stationary phase compared to the logarithmic phase. Similar to QS2-1, the Mh1128 strain produced approximately 10^4^ to 10^8^ CFU/mL of persisters tolerant to ampicillin, ceftiofur, and tetracycline during both the logarithmic and stationary phases, while the formation of persisters tolerant to florfenicol was significantly increased during the stationary phase (Figure 1O,P). These results suggest that various serotypes of *M. haemolytica* strains used in this study produce differential antibiotic tolerance in persister cell populations. Moreover, the quantity of persister cells generated by *M. haemolytica* during the stationary phase exhibited a significant increase. The findings indicate that the development of *M. haemolytica* persister cells may be predominantly associated with the metabolic level and overall vitality of the bacteria; further studies will investigate the impact of strain metabolism on the formation of persister cells.

### 3.2. The M. haemolytica Persister Cell Phenotype Is Non-Inherited

**Figure 2:**
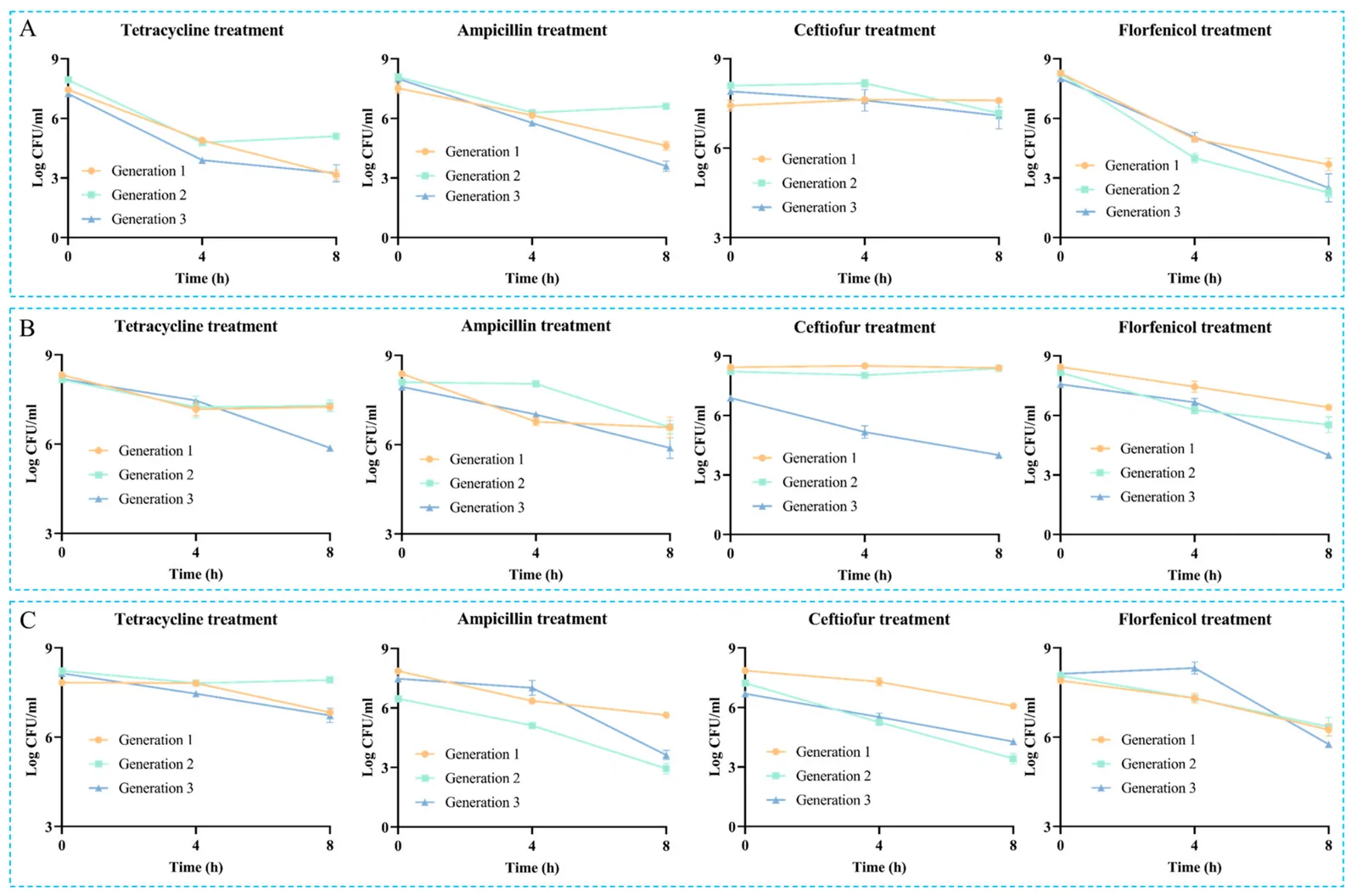
Persistence phenotype analysis of *M. haemolytica* persister cells. Surviving S19 (**A**), QS2-1 (**B**), and Mh1128 (**C**) were incubated in fresh medium overnight, then grown to the stationary phase and challenged with 50-fold MIC of tetracycline, florfenicol, or β-lactam antibiotics.

Persisters are phenotypically variant cells that differ from drug-resistant bacteria in that their drug tolerance phenotype is not inheritable. To test the heritability of antibiotic tolerance, tetracycline, ampicillin, ceftiofur, and florfenicol at 50 times the MIC were used to treat stationary-phase *M. haemolytica*, and the surviving population was repeatedly cultured for three cycles. To assess the heritability of antibiotic tolerance, stationary-phase *M. haemolytica* cultures were exposed to tetracycline, ampicillin, ceftiofur, and florfenicol at concentrations 50-fold higher than their MICs. The surviving bacterial populations were subsequently subjected to three successive rounds of culturing and treatment. The findings indicated that the antibiotic tolerance phenotype exhibited by the persister cells was not inherited by subsequent generations of *M. haemolytica* strains, irrespective of whether the serotype was A1, A2, or A6 (Figure 2 and Figure S2). From the first to the third generation, no increase in colony counts over time was observed in the presence of antibiotics. Moreover, we assessed the MIC values of antibiotics for the three generations of strains and observed no significant alterations in these MIC values (Table 2). The findings suggest that the antibiotic tolerance phenotype observed in *M. haemolytica* is non-genetically inherited.

### 3.3. Various Stressors Have Different Effects on the Formation of M. haemolytica Persisters

**Figure 3:**
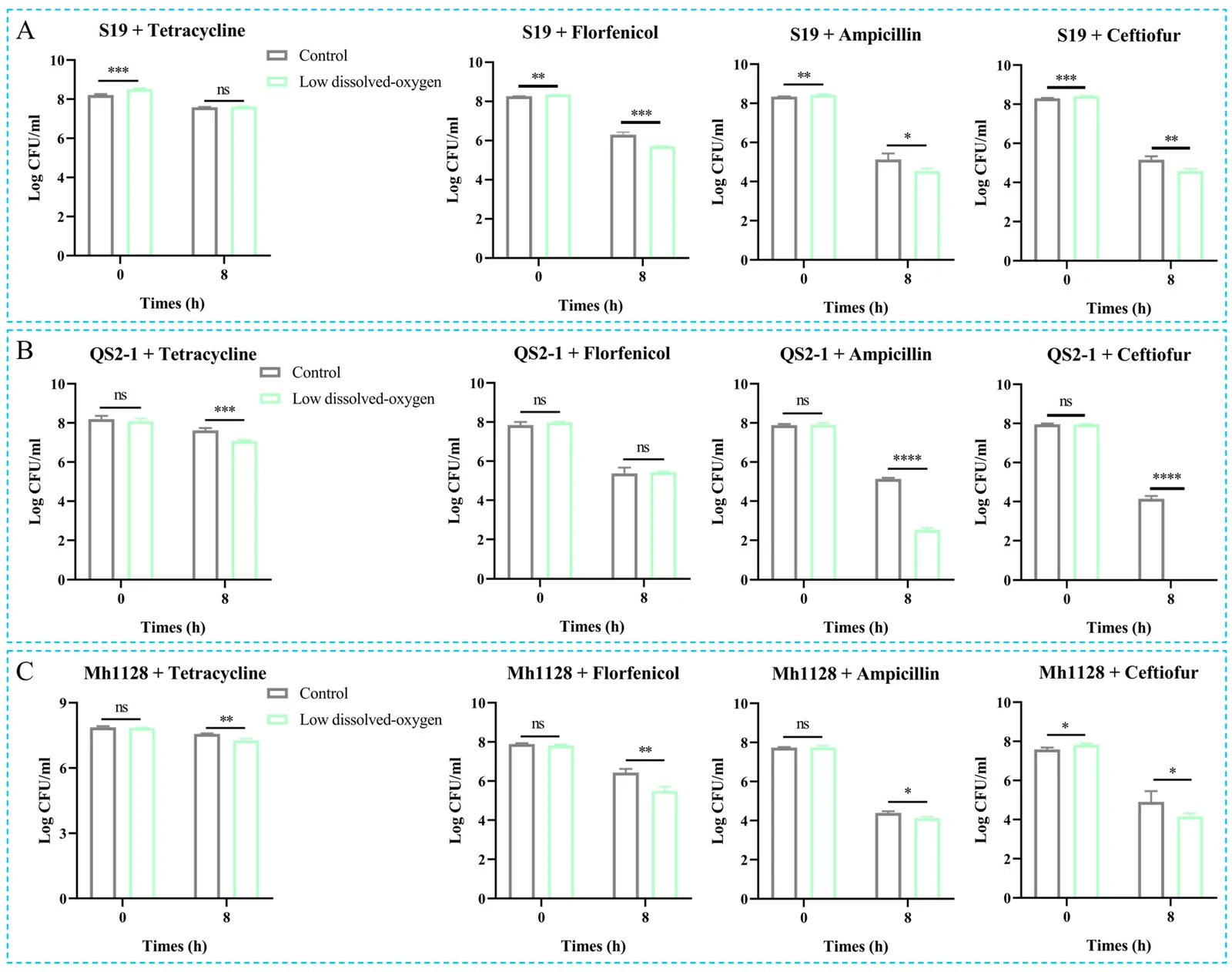
The effect of low dissolved oxygen on the formation of persisters in *M. haemolytica*. Under low dissolved oxygen conditions, stationary-phase strains S19 (**A**), QS2-1 (**B**), and Mh1128 (**C**) were treated with 50-fold MICs of antibiotics for 12 h. At 0 h and 8 h, CFUs were determined. * *p* < 0.05, ** *p* < 0.01, *** *p* < 0.001, **** *p* < 0.0001 and ns *p* > 0.05.

Bacteria are exposed to various forms of stress in both environmental settings and within the host organism. These stress factors can affect the formation of antibiotic-tolerant persisters. *M. haemolytica* bacteria live in the respiratory tract and are frequently subjected to various stressors, including hypoxic conditions, oxidative stress, and acidic environments. To investigate the ability of *M. haemolytica* to form persister cells under different stress conditions, we used three stress conditions: low dissolved oxygen, oxidative stress, and acid stress. Initially, we examined the impact of these three stress conditions on the growth of the S19, QS2-1, and Mh1128 strains. The results showed that the growth of the S19, QS2-1, and Mh1128 strains was not influenced by any of the three types of stress (Figure S3). Next, the ability of strains S19, QS2-1, and Mh1128 to form persisters under three stress conditions was further analyzed. In the S19 strain, despite the CFU count being higher in the low dissolved oxygen group compared to the control group at the initial time point (0 h), the level of persisters in the low dissolved oxygen group was significantly reduced relative to the control group following 8 h of antibiotic exposure (Figure 3A). In addition, under low dissolved oxygen conditions, the decrease in the levels of QS2-1 persisters tolerant to ampicillin and ceftiofur was more pronounced (Figure 3B). In a similar manner, low dissolved oxygen conditions also resulted in a decrease in the formation of Mh1128 persisters that are tolerant to tetracycline, florfenicol, ampicillin, and ceftiofur (Figure 3C). The results showed that under low dissolved oxygen conditions, the ability of *M. haemolytica* to form antibiotic-tolerant persisters was significantly reduced.

**Figure 4:**
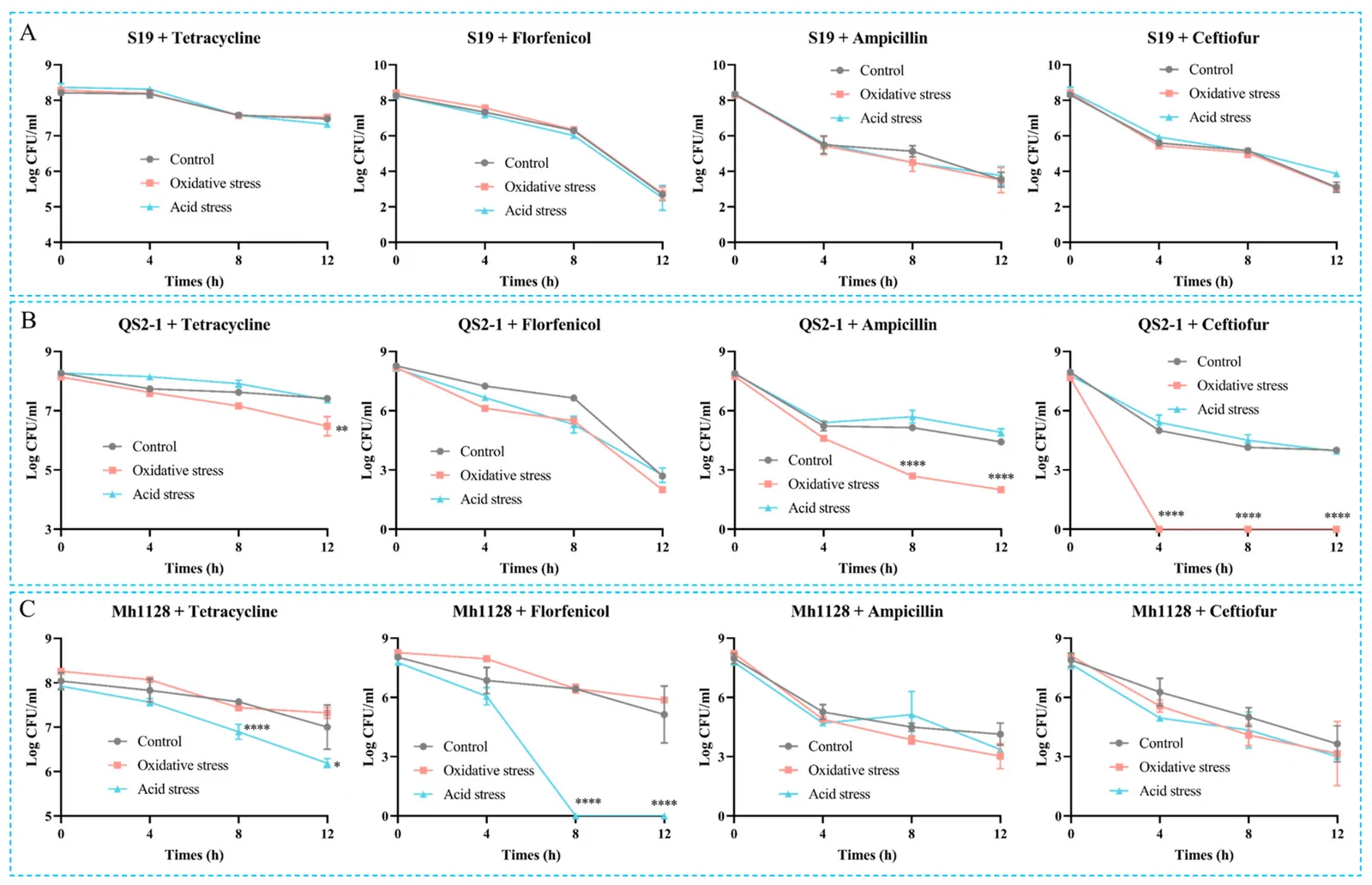
The effects of oxidative stress and acid stress on the formation of persisters in *M. haemolytica*. Under oxidative stress or acid stress conditions, stationary-phase strains S19 (**A**), QS2-1 (**B**), and Mh1128 (**C**) were treated with 50-fold MICs of antibiotics for 12 h. At the indicated time points, CFUs were determined. * *p* < 0.05, ** *p* < 0.01 and **** *p* < 0.0001.

Oxidative stress and acid stress exert distinct influences on the formation of persisters populations across various serotype strains. In the A1 serotype S19 strain, the induction of oxidative and acid stress does not influence the generation of persister cells tolerant to tetracycline, florfenicol, ampicillin, and ceftiofur (Figure 4A). However, oxidative stress led to a significant reduction in the formation of persisters tolerant to tetracycline, ampicillin, and ceftiofur in the QS2-1 strain (A2 serotype) (Figure 4B). It will especially affect the formation of QS2-1 persisters tolerant to ceftiofur. Under oxidative stress conditions, QS2-1 showed no detectable persisters after 4 h of treatment with high concentrations of ceftiofur. Acid stress reduces the formation of Mh1128 persisters that are tolerant to tetracycline and florfenicol (Figure 4C). Likewise, following exposure to acid stress conditions, Mh1128 exhibited no detectable persister cells after an 8 h treatment with elevated concentrations of florfenicol. The findings suggest that diverse stress conditions exert differential effects on the development of *M. haemolytica* persisters.

### 3.4. Exploration of Strategies for Eliminating M. haemolytica Persisters

**Figure 5:**
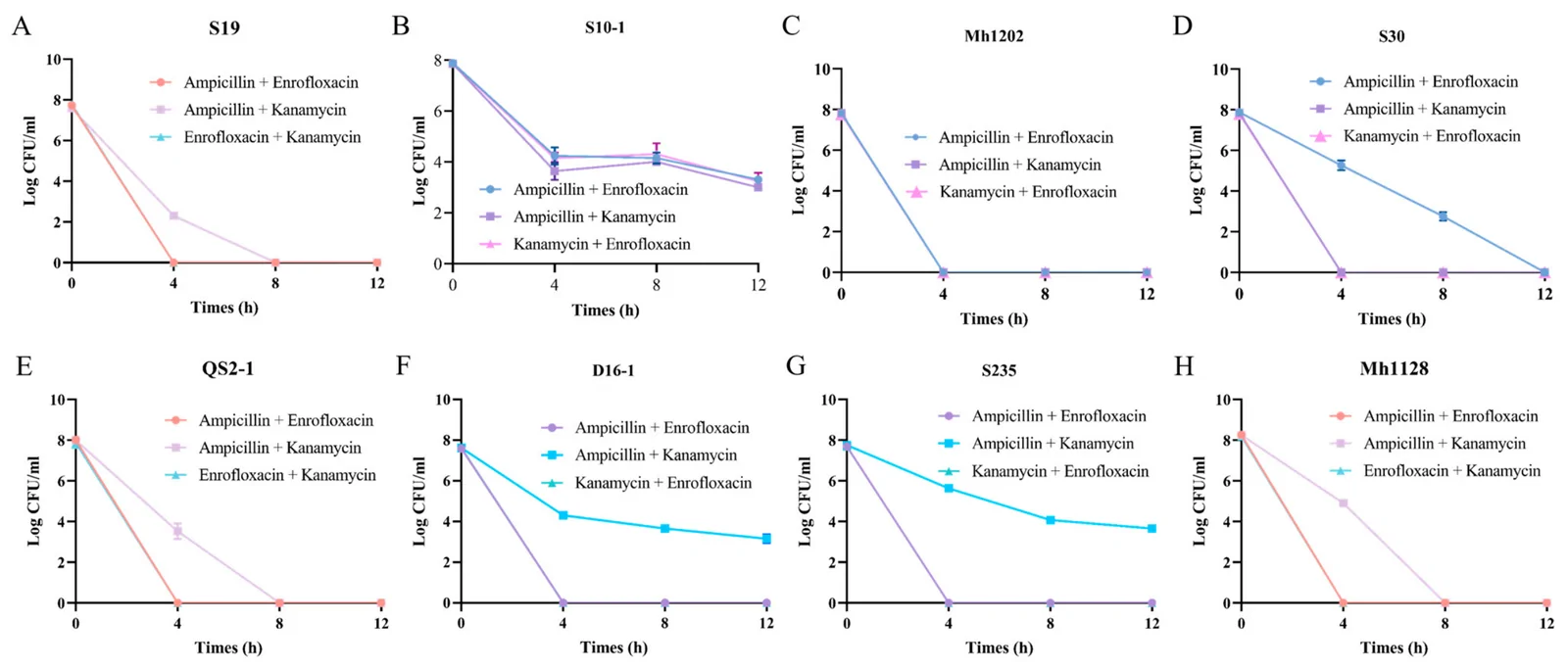
Assessment of the eradication of persisters in *M. haemolytica* by a combination of antibiotics. Stationary-phase strains S19 (**A**), S10-1 (**B**), Mh1202 (**C**), S30 (**D**), QS2-1 (**E**), D16-1 (**F**), S235 (**G**) and Mh1128 (**H**) were exposed for 12 h to combinations of antibiotics, ampicillin with enrofloxacin, ampicillin with kanamycin, and enrofloxacin with kanamycin, respectively, each administered at concentrations 50-fold greater than their MICs.

The formation of persisters is a survival strategy of bacteria under antibiotics. The eradication of persister cells constitutes a critical strategy in addressing clinically persistent and recurrent infections, as well as a key approach to preventing the emergence of pathogen resistance. To investigate methods for eliminating *M. haemolytica* persisters, we first analyzed the elimination of persisters using combinations of antibiotics with different mechanisms of action. We used three combinations of antibiotics to eliminate persister cells: Ampicillin (which inhibits cell wall synthesis) + Enrofloxacin (which inhibits DNA synthesis); Ampicillin + Kanamycin (which inhibits protein synthesis); and Enrofloxacin + Kanamycin. The results showed that all three antibiotic combinations were effective in reducing the formation of *M. haemolytica* persisters under our experimental conditions (including S19, Mh1202, S30, QS2-1, and Mh1128) (Figure 5). However, for the S10-1 strain, none of the three antibiotic combinations effectively reduced the formation of persister cells (Figure 5B). Meanwhile, for strains D16-1 and S235, although the ampicillin + enrofloxacin and kanamycin + enrofloxacin combinations effectively reduced persister formation, the ampicillin + kanamycin combination had no such effect (Figure 5F,G). These results indicate that the effectiveness of antibiotic combinations in eradicating persister cells varies significantly among different strains of *M. haemolytica*.

**Figure 6:**
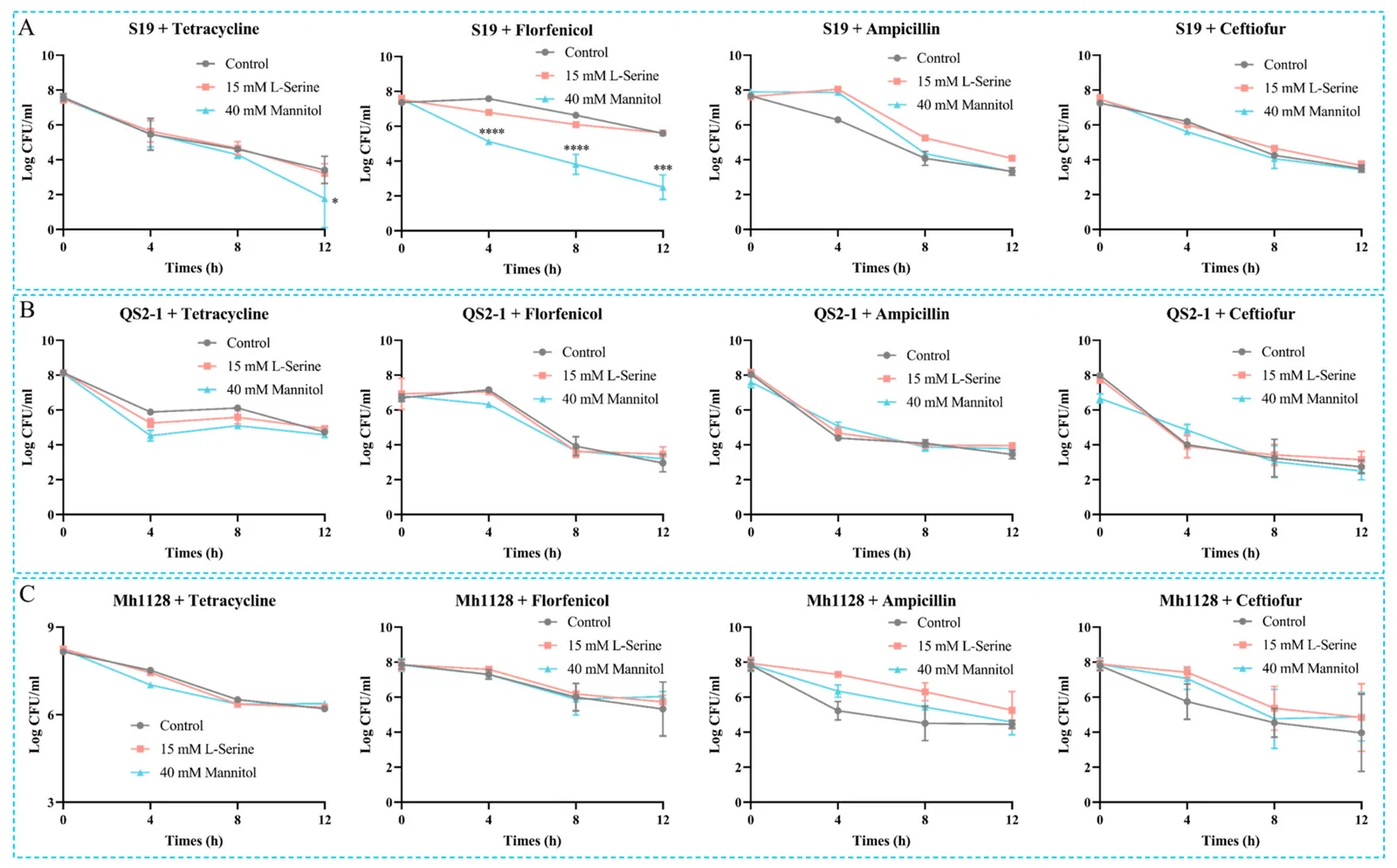
Evaluation of L-serine and mannitol for the eradication of *M. haemolytica* persisters. Under conditions supplemented with L-serine or mannitol, stationary-phase strains S19 (**A**), QS2-1 (**B**), and Mh1128 (**C**) were treated with antibiotics at 50-fold MICs for 12 h. *** *p* < 0.001 and **** *p* < 0.0001.

Previous studies have reported that L-serine can increase the sensitivity of *Escherichia coli* to ofloxacin and moxifloxacin, while promoting the killing of persister cells [44]. L-serine, as the most effective amino acid inhibitor, can inhibit bacterial growth. To investigate whether L-serine can promote the clearance of *M. haemolytica* persisters, we treated *M. haemolytica* with 15 mM L-serine. The findings demonstrated that L-serine influenced the susceptibility of strains S10-1, Mh1202, S30, and S235 to florfenicol, markedly diminishing their capacity to form persister cells (Figure S4). In contrast, L-serine did not impact persister cell formation in the other strains when exposed to antibiotics (Figure 6 and Figure S4). Prior research has demonstrated that mannitol facilitates the removal of *P. aeruginosa* persisters within biofilms [45]. Accordingly, the role of mannitol in eliminating *M. haemolytica* persisters was further investigated. Mannitol significantly reduces the formation of persisters tolerant to florfenicol in the S19, S10-1, Mh1202, S30, and S235 strains (Figure 6A and Figure S4). Nevertheless, mannitol did not exhibit a significant impact on the formation of persisters in other strains (Figure 6B,C and Figure S4D). These results provide a reference for the eradication of *M. haemolytica* persister cells.

## 4. Discussion

Beyond the acquisition of resistance genes or the occurrence of mutations, the development of antibiotic-tolerant persister populations constitutes a vital survival mechanism enabling bacteria to withstand antibiotic-induced lethality [26]. The formation of persisters significantly increases the likelihood of treatment failure, persistent infections, and recurrent infections during clinical antibiotic therapy [46]. The ability of various pathogens to form persisters has been characterized, and the formation of persisters undoubtedly poses significant challenges to clinical treatment. *M. haemolytica*, as an opportunistic pathogen commonly found in the respiratory tracts of ruminants, holds particular significance for the development of animal husbandry, especially in cattle and sheep farms [5,6]. Furthermore, given the increasingly severe antibiotic resistance among respiratory pathogens in ruminants, the ability of *M. haemolytica* to form persisters warrants attention. This study represents the first comprehensive analysis of the persister cell formation capabilities among three clinically significant serotype strains, demonstrating that the *M. haemolytica* strains tested are capable of generating persister cells even when exposed to elevated concentrations of antibiotics. In *E. coli*, key mechanisms of persister cell formation have been explored using approaches such as RNA-seq [47]. Future research could employ whole-genome sequencing and RNA sequencing of surviving subpopulations to corroborate the phenotypic traits and molecular mechanisms of persisters in *M. haemolytica*. This observation is consistent with findings reported for various Gram-positive and Gram-negative bacterial species, thereby underscoring the extensive ability of bacteria to develop persisters as a strategy to withstand antibiotic treatment. Persister formation varies among different bacterial strains and under exposure to different antibiotics [48,49]. Given the limited number of bacterial strains and antibiotics tested in the current study, future research should include a wider diversity of *M. haemolytica* strains and a broader panel of antibiotics, thereby allowing a more comprehensive evaluation of the tolerance of this species to antibiotics commonly used in clinical settings. Furthermore, as the present investigation, similar to most prior studies, is based on in vitro antibiotic tolerance assays [27,31], future work should incorporate in vivo models to examine the extent of persister cell formation by these strains within the host environment. The level of persisters formed by *M. haemolytica* in the stationary phase is higher than that in the logarithmic phase, especially for persisters tolerant to enrofloxacin and florfenicol. Elevated levels of persister cell formation during the stationary phase have been documented in various other bacterial species [31]. During the stationary phase, bacterial metabolic activity diminishes, with a substantial proportion of cells entering a dormant state. Exposure to antibiotics during this phase can facilitate the accelerated emergence of antibiotic-tolerant persister cells. Our findings further indicate that the formation of most *M. haemolytica* persisters may be associated with the bacteria’s metabolic state, though the underlying mechanism requires further investigation. In Gram-negative bacteria, the canonical pathway of persister cell formation involves the toxin of a toxin–antitoxin system acting on the bacterial replication or translation processes, thereby driving the bacteria into a dormant state that tolerates the lethal action of antibiotics [50]. In addition to reducing their own metabolism, bacteria can also enhance the transcriptional expression of certain genes, such as virulence and cell division genes [51], thereby decreasing the intracellular antibiotic concentration and conferring antibiotic tolerance. Owing to the ability of *M. haemolytica* to form persister cells upon exposure to multiple antibiotics, the mechanisms underlying persister formation will represent a central focus of future investigation.

The formation mechanism of persisters is complex, and its formation level is influenced by various environmental factors. *M. haemolytica* is a common respiratory tract bacterium that is susceptible to various stressors. During outbreaks of respiratory diseases, *M. haemolytica* encounters various environmental stressors, including hypoxic conditions, acidic environments, oxidative challenges, and osmotic stress induced by elevated salt concentrations [7,52]. Analysis revealed that under low dissolved oxygen conditions, the ability of *M. haemolytica* to form persisters was significantly reduced. Nonetheless, dissolved oxygen levels were not directly measured in this study, which may introduce some uncertainty in evaluating the severity of low dissolved oxygen stress. Future investigations should therefore incorporate monitoring systems to precisely verify these conditions. In contrast, *Mycobacterium smegmatis* exhibits higher levels of persistent bacteria formation under low dissolved oxygen conditions [37]. One mechanism by which antibiotics exert bactericidal effects involves the generation of reactive oxygen species; consequently, the attenuation of reactive oxygen species levels may enhance bacterial survival. The finding that low dissolved oxygen inhibits the formation of persisters in this study indicates that the mechanism of persister formation in *M. haemolytica* exhibits significant variation. In oxidative stress conditions, only QS2-1 exhibited a significant reduction in the formation of β-lactam antibiotic-tolerant persisters, while no significant changes were observed in the other strains or antibiotics. The effects of oxidative stress on the formation of persisters in *M. haemolytica* are related to both the bacterial strain and the type of antibiotic, which differs from findings in *S. aureus* [43]. Additionally, the formation of tetracycline- and florfenicol-tolerant persisters in *M. haemolytica* Mh128 is influenced by acid stress. The formation of antibiotic-tolerant persisters varies significantly among different strains, indicating the complexity of the mechanism underlying the formation of persisters in *M. haemolytica*. Given that the capacity for persister formation may vary considerably among different strains due to differences in genetic background, clinical origin, and prior antimicrobial exposure [48,49], the results presented in this study should be regarded as a preliminary assessment derived from representative strains of the three major serotypes. To validate and extend the findings of this study, future investigations should incorporate a larger number of strains per serotype, including isolates from diverse geographic regions, clinical sources, and with varying histories of antimicrobial exposure.

Due to the significance of persisters in the development of bacterial resistance and their complex formation mechanisms. The advancement of innovative antimicrobial treatments aimed at inhibiting persister formation has increasingly been recognized as a promising approach for managing bacterial infections [30,53]. Persister cells can be eradicated through two principal strategies: suppressing their formation, or triggering the resuscitation of dormant persisters to restore antibiotic susceptibility. Both approaches hold promise for reducing the incidence of persistent and recurrent infections. Numerous studies have investigated the eradication of persisters, with a particular focus on enhancing their susceptibility to antibiotics [37,45]. These studies primarily involve altering the intracellular environment and metabolic processes of bacteria by adding chemicals, thereby increasing their susceptibility to antibiotics and enhancing the probability of killing persisters [44,45,54]. The combination of antibiotics is particularly common and important in the treatment of clinical bacterial infections. The use of combination antibiotic therapy to eradicate persisters has the potential to optimize the treatment protocol and decrease associated healthcare expenses. By combining antibiotics with different mechanisms of action, we achieve a rapid reduction in the formation of some *M. haemolytica* persisters under our experimental conditions. The approach of eradicating *M. haemolytica* persister cells via the combined administration of antibiotics holds potential for implementation in the clinical management of ruminant livestock. This approach will provide valuable insights for developing strategies to eradicate persister cells. The clearance of persister cells has been investigated in mouse infection models for both Staphylococcus aureus and Escherichia coli [55,56]. Future studies should focus on validating the in vivo applicability of combination antibiotic regimens using animal models. However, the addition of L-serine and mannitol yielded poor results, differing from those observed in *E. coli* and *P. aeruginosa*. These studies suggest that the mechanisms underlying persister formation differ across bacterial species and exhibit considerable complexity. Further exploration is needed to understand the mechanisms underlying the formation of persisters and the strategies for their clearance across different pathogens.

## 5. Conclusions

Our study shows that the ruminant respiratory pathogen *M. haemolytica* can form persister cells under multiple antibiotics. The development of antibiotic-tolerant persisters is predominantly associated with the growth state of *M. haemolytica*. Moreover, the formation of persisters across different serotype strains is affected by diverse stress factors. Meanwhile, our findings indicate that combination antibiotic therapy is effective in reducing the formation of *M. haemolytica* persisters. This study will provide a foundation for investigating the mechanisms underlying the formation of persisters in the respiratory tracts of ruminant animals.

## Figures and Tables

**Table 1:** The MICs of strains to antibiotics.

Antimicrobial	MIC (μg/mL)							
	S19	S10-1	Mh1202	S30	D16-1	S235	QS2-1	Mh1128
Tetracycline	1	0.4	0.8	0.4	0.8	0.8	0.5	1
Terramycin	<0.25	0.8	1.6	0.8	1.6	3.2	0.5	<0.25
Ampicillin	<0.25	3.2	1.6	0.8	3.2	3.2	2	<0.25
Ceftiofur	<0.25	1.6	0.8	0.8	0.4	3.2	<0.25	<0.25
Florfenicol	0.5	0.8	0.8	0.8	0.8	0.8	0.5	0.5
Enrofloxacin	<0.25	0.4	0.8	0.4	0.05	0.05	<0.25	<0.25

**Table 2:** The MICs of different generations to antibiotics.

**Generations**	**S19**			
	**Tetracycline**	**Ceftiofur**	**Ampicillin**	**Florfenicol**
Generation 1	0.25 μg/mL	<0.25 μg/mL	0.5 μg/mL	0.25 μg/mL
Generation 2	0.5 μg/mL	<0.25 μg/mL	0.5 μg/mL	0.5 μg/mL
Generation 3	0.5 μg/mL	<0.25 μg/mL	0.25 μg/mL	0.25 μg/mL
**Generations**	**S10-1**			
	**Tetracycline**	**Ceftiofur**	**Ampicillin**	**Florfenicol**
Generation 1	0.4 μg/mL	0.8 μg/mL	3.2 μg/mL	0.8 μg/mL
Generation 2	0.4 μg/mL	0.8 μg/mL	3.2 μg/mL	0.8 μg/mL
Generation 3	0.4 μg/mL	0.8 μg/mL	3.2 μg/mL	0.8 μg/mL
**Generations**	**Mh1202**			
	**Tetracycline**	**Ceftiofur**	**Ampicillin**	**Florfenicol**
Generation 1	1.6 μg/mL	1.6 μg/mL	1.6 μg/mL	0.4 μg/mL
Generation 2	1.6 μg/mL	1.6 μg/mL	1.6 μg/mL	0.4 μg/mL
Generation 3	1.6 μg/mL	1.6 μg/mL	1.6 μg/mL	0.4 μg/mL
**Generations**	**S30**			
	**Tetracycline**	**Ceftiofur**	**Ampicillin**	**Florfenicol**
Generation 1	0.8 μg/mL	1.6 μg/mL	1.6 μg/mL	0.8 μg/mL
Generation 2	0.8 μg/mL	1.6 μg/mL	1.6 μg/mL	0.8 μg/mL
Generation 3	0.8 μg/mL	1.6 μg/mL	1.6 μg/mL	0.8 μg/mL
**Generations**	**D16-1**			
	**Tetracycline**	**Ceftiofur**	**Ampicillin**	**Florfenicol**
Generation 1	0.4 μg/mL	0.8 μg/mL	3.2 μg/mL	1.6 μg/mL
Generation 2	0.4 μg/mL	0.8 μg/mL	3.2 μg/mL	0.8 μg/mL
Generation 3	0.4 μg/mL	0.4 μg/mL	3.2 μg/mL	0.8 μg/mL
**Generations**	**S235**			
	**Tetracycline**	**Ceftiofur**	**Ampicillin**	**Florfenicol**
Generation 1	0.4 μg/mL	1.6 μg/mL	0.8 μg/mL	0.4 μg/mL
Generation 2	0.2 μg/mL	1.6 μg/mL	0.8 μg/mL	0.4 μg/mL
Generation 3	0.4 μg/mL	1.6 μg/mL	0.8 μg/mL	0.4 μg/mL
**Generations**	**QS2-1**			
	**Tetracycline**	**Ceftiofur**	**Ampicillin**	**Florfenicol**
Generation 1	0.25 μg/mL	<0.25 μg/mL	1 μg/mL	0.25 μg/mL
Generation 2	0.25 μg/mL	<0.25 μg/mL	1 μg/mL	0.25 μg/mL
Generation 3	0.25 μg/mL	<0.25 μg/mL	0.5 μg/mL	0.25 μg/mL
**Generations**	**Mh1128**			
	**Tetracycline**	**Ceftiofur**	**Ampicillin**	**Florfenicol**
Generation 1	1 μg/mL	<0.25 μg/mL	0.25 μg/mL	1 μg/mL
Generation 2	1 μg/mL	<0.25 μg/mL	0.5 μg/mL	0.5 μg/mL
Generation 3	0.5 μg/mL	<0.25 μg/mL	0.5 μg/mL	0.5 μg/mL

## Data Availability

All data for this study are available within the article.

## Supplementary Materials

The following supporting information can be downloaded at https://www.mdpi.com/article/10.3390/ani16162610/s1: Figure S1. Identification of *M. haemolytica* and Serotypes. (A) Identification of serotype A1. (B) Identification of serotype A2. (C) Identification of serotype A6. (D) Identification of *M. haemolytica*. M: Marker; P: Positive; N: Negative. Figure S2. Persistence phenotype analysis of *M. haemolytica* persister cells. Surviving S10-1 (A), Mh1202 (B), S30 (C), D16-1 (D), and S235 (E) were incubated in fresh medium overnight, then grown to the stationary phase and challenged with 50-fold MIC of tetracycline, florfenicol, or β-lactam antibiotics. Figure S3. Evaluation of the effects of different stressors on the growth of *M. haemolytica*. Under conditions of low dissolved oxygen, oxidative stress, or acid stress, determine the CFUs at the indicated time points. Figure S4. Evaluation of L-serine and mannitol for the eradication of *M. haemolytica* persisters. Under conditions supplemented with L-serine or mannitol, stationary-phase strains S10-1 (A), Mh1202 (B), S30 (C), D16-1 (D), and S235 (E) were treated with antibiotics at 50-fold MICs for 12 h. Table S1. All strains used in this study. Table S2. All primers used in this study.
