# Supplementary material for: Characterization of Antibiotic-Tolerant Persister Cells in Mannheimia haemolytica
Source: Animals (Basel). 2026 Aug 20;16(16):2610. doi: 10.3390/ani16162610 (PMC13508971; doi:10.3390/ani16162610)
Supplement: Supplementary file 1 [file animals-16-02610-s001.zip › Figure S1.pdf]

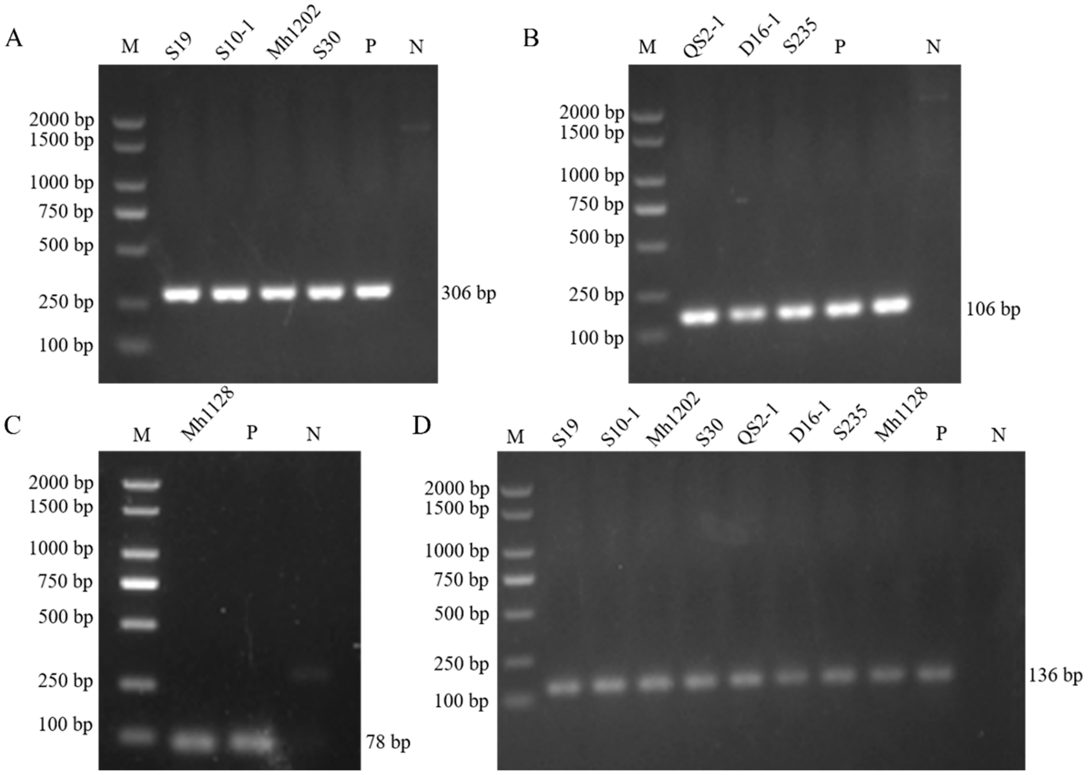

Figure S1. Identification of *M. haemolytica* and Serotypes. (A) Identification of serotype A1. (B) Identification of serotype A2. (C) Identification of serotype A6. (D) Identification of *M. haemolytica*. M: Marker; P: Positive; N: Negative.
