# Supplementary material for: Characterization of Antibiotic-Tolerant Persister Cells in Mannheimia haemolytica
Source: Animals (Basel). 2026 Aug 20;16(16):2610. doi: 10.3390/ani16162610 (PMC13508971; doi:10.3390/ani16162610)
Supplement: Supplementary file 1 [file animals-16-02610-s001.zip › Figure S2.pdf]

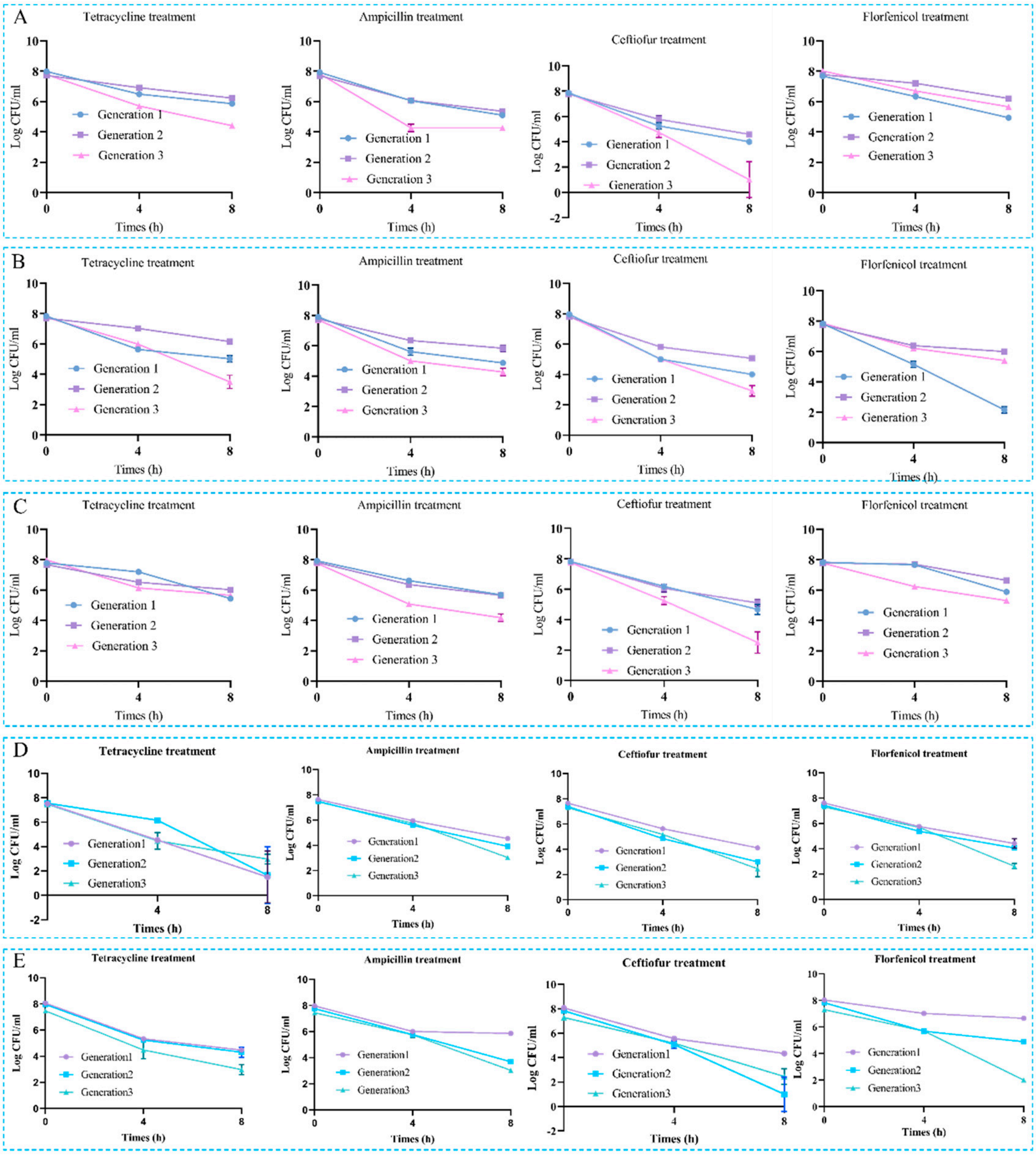

Figure S2. Persistence phenotype analysis of *M. haemolytica* persister cells. Surviving S10-1 (A), Mh1202 (B), S30 (C), D16-1 (D), and S235 (E) were incubated in fresh medium overnight, then grown to the stationary phase and challenged with 50-fold MIC of tetracycline, florfenicol, or β-lactam antibiotics.
