# Supplementary material for: Characterization of Antibiotic-Tolerant Persister Cells in Mannheimia haemolytica
Source: Animals (Basel). 2026 Aug 20;16(16):2610. doi: 10.3390/ani16162610 (PMC13508971; doi:10.3390/ani16162610)
Supplement: Supplementary file 1 [file animals-16-02610-s001.zip › Figure S3.pdf]

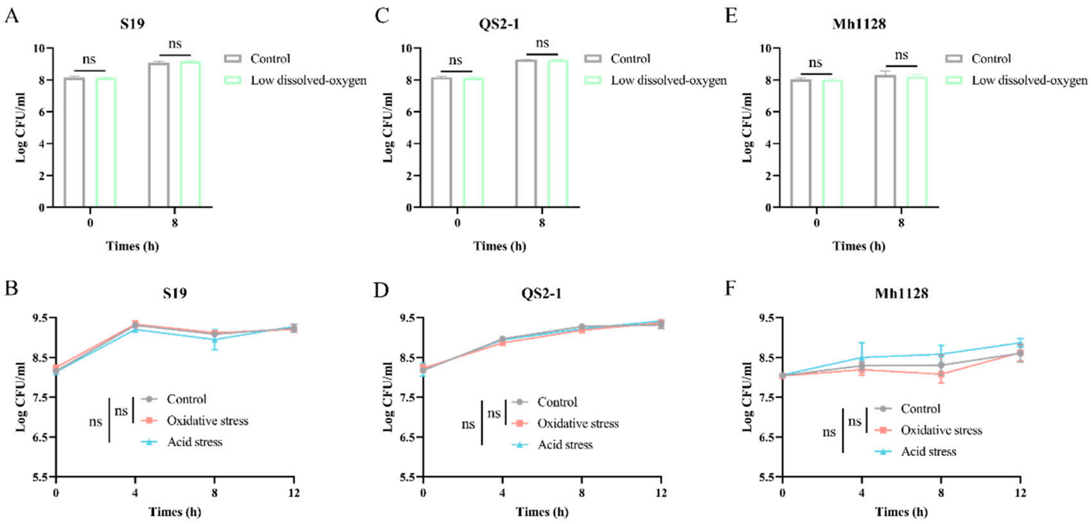

Figure S3. Evaluation of the effects of different stressors on the growth of *M. haemolytica*. Under conditions of low dissolved oxygen, oxidative stress, or acid stress, determine the CFUs at the indicated time points.
