# Supplementary material for: Characterization of Antibiotic-Tolerant Persister Cells in Mannheimia haemolytica
Source: Animals (Basel). 2026 Aug 20;16(16):2610. doi: 10.3390/ani16162610 (PMC13508971; doi:10.3390/ani16162610)
Supplement: Supplementary file 1 [file animals-16-02610-s001.zip › Figure S4.pdf]

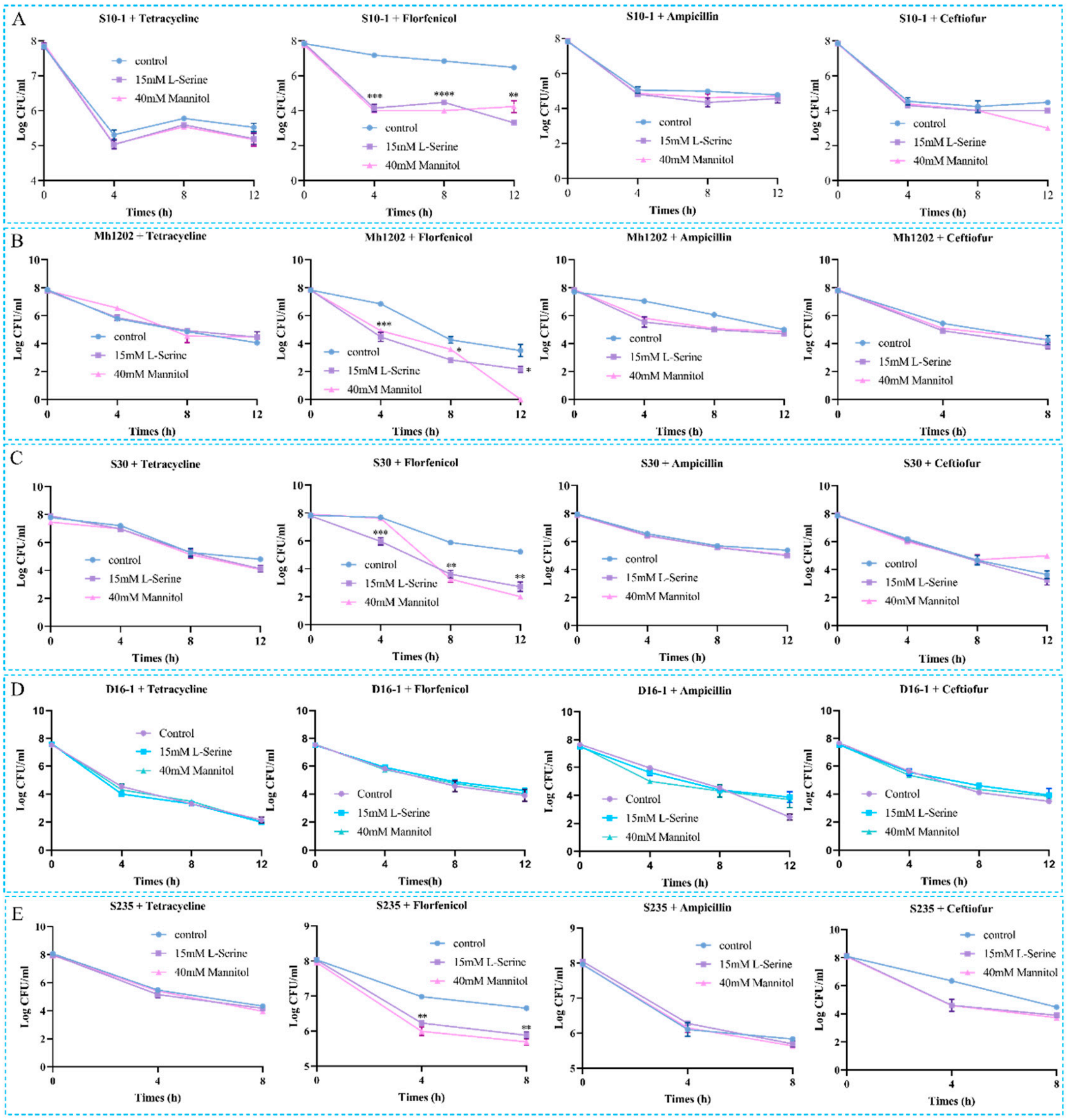

Figure S4. Evaluation of L-serine and mannitol for the eradication of *M. haemolytica* persisters. Under conditions supplemented with L-serine or mannitol, stationary-phase strains S10-1 (A), Mh1202 (B), S30 (C), D16-1 (D), and S235 (E) were treated with antibiotics at 50-fold MICs for 12 hours.
