# Supplementary material for: Characterization of Antibiotic-Tolerant Persister Cells in Mannheimia haemolytica
Source: Animals (Basel). 2026 Aug 20;16(16):2610. doi: 10.3390/ani16162610 (PMC13508971; doi:10.3390/ani16162610)
Supplement: Supplementary file 1 [file animals-16-02610-s001.zip › Table S1.pdf]

Table S1. All strains used in this study.

Bacterial strains	Description	Host	Reference
S19	Serotype A1 wild type	Sheep	Collected in our lab
S10-1		Sheep	Collected in our lab
Mh1202		Sheep	Collected in our lab
S30		Sheep	Collected in our lab
QS2-1	Serotype A2 wild type	Sheep	Collected in our lab
D16-1		Sheep	Collected in our lab
S235		Sheep	Collected in our lab
Mh1128	Serotype A6 wild type	Goat	Collected in our lab
