# Supplementary material for: Characterization of Antibiotic-Tolerant Persister Cells in Mannheimia haemolytica
Source: Animals (Basel). 2026 Aug 20;16(16):2610. doi: 10.3390/ani16162610 (PMC13508971; doi:10.3390/ani16162610)
Supplement: Supplementary file 1 [file animals-16-02610-s001.zip › Table S2.pdf]

Table S2. All primers used in this study.

Primers	Sequences	Size (bp)
MropB-F	AACACATAAACGCCGTATCTCG	136
MropB-R	GATATTCGGGCCTTCAGGA	
MhA1-F	CATTTCCTTAGGTTCAGC	306
MhA1-R	CAAGTCATCGTAATGCCT	
MhA2-F	GGCATATCCTAAAGCCGT	106
MhA2-R	AGAATCCACTATTGGGCACC	
MhA6-F	TGAGAATTTCGACAGCACT	78
MhA6-R	ACCTTGGCATATCGTACC	
